# Chronic Pain Induced by Social Defeat Stress in Juvenile Mice Depends on TLR4

**DOI:** 10.3390/cells14050350

**Published:** 2025-02-27

**Authors:** Julia Borges Paes Lemes, Alisa Panichkina, Kaue Franco Malange, Carlos E. Morado-Urbina, Sara Anna Dochnal, Saee Jadhav, Maksim Dolmat, Marco Pagliusi, Juliana M. Navia-Pealez, Maripat Corr, Yury I. Miller, Tony L. Yaksh

**Affiliations:** 1 Department of Anesthesiology, University of California, San Diego, CA 92093, USA; apanichk@ucsd.edu (A.P.); kfrancomalange@health.ucsd.edu (K.F.M.); cmoradourbina@health.ucsd.edu (C.E.M.-U.); sdochnal@health.ucsd.edu (S.A.D.); syjadhav@ucsd.edu (S.J.); tyaksh@health.ucsd.edu (T.L.Y.); 2 Department of Chemical and Nano Engineering, University of California, San Diego, CA 92093, USA; mdolmat@ucsd.edu; 3 Department of Pharmacology, Ribeirão Preto Medical School, University of São Paulo, Sao Paulo 14049-900, Brazil; mpagliusi@usp.br; 4 Department of Pharmacology and Physiology, Saint Louis University, Saint Louis, MO 63104, USA; juliana.navia@health.slu.edu; 5 Department of Medicine, University of California, San Diego, CA 92093, USA; mpcorr@health.ucsd.edu (M.C.); yumiller@health.ucsd.edu (Y.I.M.)

**Keywords:** pain, social stress, TLR4, spinal cord, dorsal root ganglia

## Abstract

A significant portion of adolescents suffer from mental illnesses and persistent pain due to repeated stress. The components of the nervous system that link stress and pain in early life remain unclear. Prior studies in adult mice implicated the innate immune system, specifically Toll-like receptors (TLRs), as critical for inducing long-term anxiety and pain-like behaviors in social defeat stress (SDS) models. In this work, we investigated the pain and anxiety behavioral phenotypes of wild-type and TLR4-deficient juvenile mice subjected to repeated SDS and evaluated the engagement of TLR4 by measuring dimerization in the spinal cord, dorsal root ganglia, and prefrontal cortex. Male juvenile (4-week-old) mice (C57BL/6J or *Tlr4^-/-^*) underwent six social defeat sessions with adult aggressor (CD1) mice. In WT mice, SDS promotes chronic mechanical allodynia and thermal hyperalgesia assessed via von Frey testing and the Hargreaves test, respectively. In parallel, the stressed WT mice exhibited transient anxiety-like behavior and long-lasting locomotor activity reduction in the open-field test. *Tlr4^-/-^*-stressed animals were resistant to the induction of pain-like behavior but had a remnant of anxious behavior, spending less time in the center of the arena. In WT SDS, there were concordant robust increases in TLR4 dimerization in dorsal root ganglia macrophages and spinal cord microglia, indicating TLR4 activation. These results suggest that the chronic pain phenotype and locomotor impairment induced by SDS in juvenile mice depends on TLR4 engagement evidenced by dimerization in immune cells of the dorsal root ganglia and spinal cord.

## 1. Introduction

Stress and pain are multifactorial constructs that are linked at several levels. Both activate similar pathways of the nervous system, such as the corticolimbic system, hippocampus, and prefrontal cortex, and can be affected by genetic factors and psychosocial triggers (fears, emotional distress, beliefs, goals, and threats) [1,2,3]. In acute conditions, stress may act as a protective response, inducing systemic cortisol release that provides energy to ‘escape’ from the stressor (a fight or flight state) [4]. However, stress becomes harmful when it occurs repeatedly for an extended period, leading to dysregulation in the neuroendocrine and immune systems [1]. Evidence shows that chronic stress is a major trigger for several mental disorders, such as anxiety and depression, and when it occurs in childhood or adolescent years, the individual becomes predisposed to the development of psychopathologies and pain sensitivity associated with increases in circulating inflammatory mediators released during stressful events [5]. In patients with depression, an increase in cytokines in the peripheral blood was detected, which was also related to the participation of endogenous damage-associated molecular patterns (DAMPs) [6].

Preclinical models of physical and psychological stress have been used in rodents by applying unpleasant stressors such as repeated restraint, forced swims, sleep deprivation, maternal separation, and social defeat stress (SDS) [7]. The SDS model involves the resident–intruder paradigm in which a submissive mouse intruder is physically and sensorially exposed to an aggressor animal (resident) [8]. SDS was shown to promote behavioral alterations in adult mice, including anxiety, anhedonia, and social avoidance [9]. Interestingly, SDS also triggers a widespread chronic pain phenotype, mimicking a condition similar to human fibromyalgia [9]. At present, there is an increasing interest in the study of pain phenotypes evoked by SDS in juvenile mice, given some clinical findings linking children and adolescent bullying with mental disorders and increased susceptibility to develop chronic pain in adulthood [10,11].

Our group has focused on studying the potential mechanisms and molecules for addressing chronic pain. In such an effort, Toll-like receptor 4 (TLR4) has emerged as a key factor for neuronal sensitization [12,13,14,15]. These receptors are highly expressed in neural tissues, particularly in neuraxial microglia and dorsal root ganglion macrophages [16], and are demonstrated to participate in initiating and maintaining pain states, such as peripheral neuropathic pain induced by chemotherapy [17] and arthritic pain [18]. Despite this, there are many gaps to be filled regarding the mechanisms involved in the hyperexcitability of pain pathways as a result of chronic social stress, especially at early stages. Here, using social defeat stress (SDS), we investigated the chronic pain and anxiety-like behavior phenotype in juvenile male mice and the contribution of TLR4 activity in the dorsal root ganglia, spinal cord, and prefrontal cortex in the development of persistent pain caused by social stress.

## 2. Materials and Methods

### 2.1. Animals

Male C57BL/6J mice at four to five weeks of age were used as the experimental mice and randomly divided into stressed (SDS) or non-stressed (no-SDS) groups, and twelve-week-old male CD1 retired breeders were used as aggressors (purchased from Jackson laboratory^©^ or ENVIGO^©^, San Diego, CA, USA). They were maintained on isolated ventilated racks in a 12:12 h light or dark cycle with controlled humidity and temperature (22 °C). Food and water were available ad libitum. *Tlr4^-/-^* mice (TLR4-KO) were a gift from Dr. S. Akira (Osaka University, Osaka, Japan) and backcrossed onto the C57BL/6 background for ten generations [19]. Animal handling and behavioral experiments were performed as per the International Association for the Study of Pain (IASP) guidelines for using animals in pain research. The study was conducted in accordance with the rules established by the Institutional Animal Care and Use Committee (IACUC) of the University of California, San Diego, USA. All animal procedures performed in this study were approved by the IACUC (#S00137M, 19 July 2023).

### 2.2. Social Defeat Stress (SDS)

Social defeat stress is an ethologically relevant and widely used stress model to mimic the symptomatology of human depression and chronic pain and is based on a resident–intruder paradigm [8]. We first screened several CD1 mice to categorize aggressive behavior. For this, we used a separate group of C57BL/6J mice as screeners. The screening consisted of 3 consecutive daily sessions of 3 min of physical contact between a CD1 with a C57BL/6 mouse who was placed in the aggressor cage. A new screener mouse was placed in the CD1 cage in each session. The latency of the CD1 mouse’s first attack and the number of assaults against the screener were monitored and recorded following previous protocol standards [20]. Intermediated CD1 aggressive behavior was selected to perform the SDS protocol.

SDS protocol consisted of 6 physical sessions of 10 min between a C57BL/6J experimental mouse and a CD1 (resident) mouse, which were previously selected. They were maintained in the same cage for ten days and separated by a clear perforated plexiglass divider, allowing only sensorial contact (Supplementary Figure S1). The physical sessions were split every two days (protocol days 1, 2, 5, 6, 9, and 10). The control group had no physical contact with the CD1 aggressor or other mice, being paired for ten days with another naive C57BL/6 mouse separated by a perforated plexiglass divider (Supplementary Figure S1). For acclimation, the divider was placed two days before the first stress session in the aggressor and control cages. After the end of the SDS protocol, on day 11, all experimental mice (SDS and no-SDS) were single-housed for further behavioral assessment.

### 2.3. Mechanical and Thermal Threshold Evaluation

The mechanical threshold was measured by von Frey filaments (Stoelting Co., Wood Dale, IL, USA) using the up–down method [21]. Different gauges or stiffness filaments (range from 2.44 to 4.31; 0.02–2.00 g) were applied to the plantar surface of both hind paws, and the withdrawal response was registered. Before the initiation of mechanical stimulation, mice were placed in clear, plastic, bottomless cages over a wire mesh surface and acclimated for 1 h. Mechanical values for the left and right hind paws were measured and averaged to produce a single data point per measurement day.

Thermal hyperalgesia was evaluated by a Hargreaves-type testing device (Department of Anesthesiology, University of California, San Diego, CA, USA) [22]. The device consists of a box with a glass surface on which the mice rest in plexiglass cubicles for 1 h. A projection bulb is positioned below the glass surface, and when activated, it projects a focus heat light at 36–43 °C. The thermal stimulus is applied to both hind paws, and latency is defined as the time required for the mouse to produce a brisk paw withdrawal as detected by photodiode motion sensors that stop the timer and terminate the thermal stimuli. Von Frey and Hargreaves tests were performed before the stress session (day 0) and on days 7, 11, and 21 of the SDS protocol. The investigator, blinded to the treatment, conducted both behavioral tests from 9:00 a.m. to 5:00 p.m.

### 2.4. Open-Field and Light-And-Dark Tests

Mice were subjected to the open-field test to evaluate locomotor activity and anxiety-like behavior. The test consisted of recording the mouse freely moving in an arena (40 cm × 40 cm × 40 cm; 800 lx) for 10 min [23]. Specialized software (Ethovision 3.1, Noldus Information Technology BV, Wageningen, The Netherlands) was used to assess the real-time activity of each animal (nose, center of the body, and tail base). Each mouse was tested individually in a separate arena; 4 arenas were used per experiment. The parameters measured were as follows: (1) number of entries to the center, (2) time spent in the center, (3) number of entries to the corners, (4) time spent in the corners, (5) total distance traveled, (6) body mobility, (7) velocity, and (8) maximum acceleration.

The light-and-dark test was performed in an apparatus with two equally sized interconnected compartments (90 cm × 90 cm × 165 cm) [24]. One compartment was illuminated with 7000 lux white light, and the other was painted dark and covered with an opaque lid. Mice started in the dark compartment and stayed for 10 min in the arena. The video tracker system (Ethovision 3.1, Noldus Information Technology BV, Wageningen, The Netherlands) registered the latency for the first entry into the light side and the time spent. Both behavioral tests were conducted on days 10 and 17 of the protocol between 9:00 a.m. and 5:00 p.m. Before each test, animals were placed in the room for 60 min for acclimation.

### 2.5. Immunostaining

Animals were euthanized eleven days after the last session of stress, i.e., day 22 of the protocol. The spinal cord (lumbar region), dorsal root ganglia (L3, L4, and L5, right and left), and prefrontal cortex were harvested and used for flow cytometry assays or immunofluorescence. To perform flow cytometry assays, the tissues were dissociated using the Neural Tissue Dissociation Kit (Miltenyi Biotec, San Diego, CA, USA, #130.092.628), following the manufacturer’s protocol. A debris removal solution (Kit Miltenyi Biotec, San Diego, CA, USA #130.109.398) was applied to the spinal cord and prefrontal cortex samples to eliminate debris. Single-cell suspensions were incubated for 45 min on ice with the following antibodies: APC-Cy7 live/dead Ghost dye (#18452, Cell Signaling, Danvers, MA, USA) or 540 live/dead Ghost dye (#72086, Cell Signaling, Danvers, MA, USA); PC7-conjugated TLR4/MD2 (#MTS510; ThermoFisher, Waltham, MA, USA); APC-conjugated total TLR4 (#SA15-21; Biolegend, San Diego, CA, USA); 488-conjugated F4-80 (#123110; Biolegend, San Diego, CA, USA); and PercpCy 5.5-conjugated CD11b (#101228; Biolegend, San Diego, CA, USA). After staining, cells were washed and analyzed using a CytoFLEX flow cytometer (Beckman Coulter, Brea, CA, USA). To calculate TLR4 dimers, the ratio of the geometric mean fluorescence intensity of the TLR4/MD2 monomer antibody over the total TLR4 antibody was calculated: TLR4 dimers % = 100 − (TLR4 monomer gMean × %)/(TLR4 total gMean × %) × (100%)/(highest TLR4 ratio) [12,25].

The prefrontal cortex (PFC) was dissected post-fixed in a solution containing 4% formaldehyde in 0.01M PBS for 24 h and cryoprotected using 30% sucrose until the tissue sank into the solution. Using cryocut in a Leica cryostat, 30 mm slices were cut and stored in an antifreeze solution at –20 °C. After all the PFCs were cut, 5–6 slices from each sample were processed as free-floating sections, rinsed in 0.01M PBS, and blocked using 2% BSA for 60 min at room temperature. Next, the sections were incubated overnight with an anti-Iba1 antibody (1:500, Wako,#019-19741, Richmond, VA) at room temperature. The secondary antibody goat Anti-rabbit Alexa 488 (1:1000, Thermo Fisher #A11034, Waltham, MA, USA) was applied for 1 h. Sections were mounted in superfrost plus slides and coverslipped with ProLong™ Gold Antifade Mounting (Thermo Fisher, #P36935, Waltham, MA, USA). Z-stack imaging from three different ROIs of a section and three sections from each animal were executed using a Leica TCS SP5 confocal microscope with LAS AF version 2.7.3.9723 software. For quantification, the images were transformed into maximum projection, and a blind experimenter manually counted the number of positive cells for Iba1.

### 2.6. Statistical Analysis

Statistical analyses were performed in GraphPad Prism v10 (GraphPad Software Inc., La Jolla, CA, USA). Data are expressed as mean ± Standard Error of the Mean (SEM), and statistical differences were determined using one-way or two-way analyses of variance (ANOVAs) followed by Bonferroni’s multiple comparison test. Means were considered significantly different when *p* < 0.05. The Institutional Animal Care and Use Committee of the University of California, San Diego, approved all animal experimental procedures performed in this work (IACUC—protocol number #S00137M, 19 July 2023).

## 3. Results

### 3.1. SDS Induces Persistent Mechanical Allodynia and Thermal Hyperalgesia

**Figure 1:**
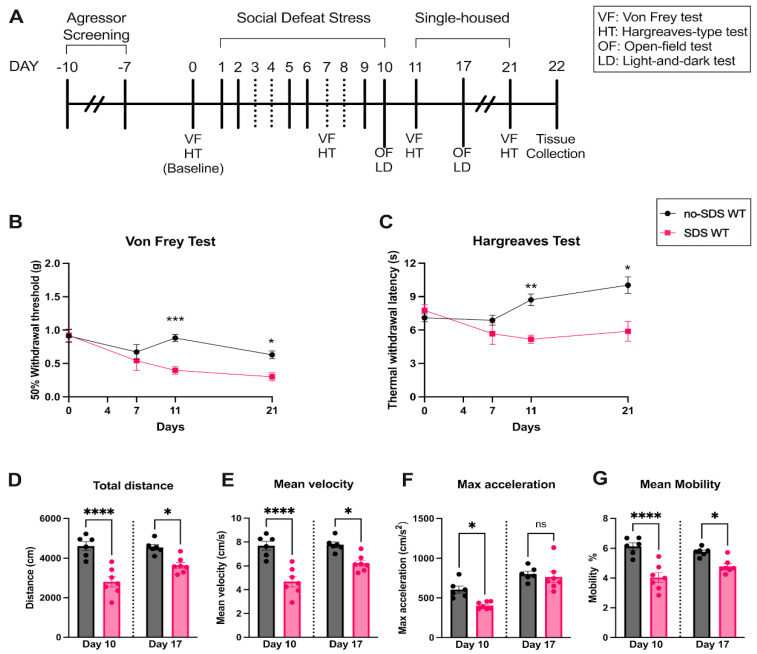
SDS promotes pain phenotype and reduction in locomotor activity. (**A**) Experimental timeline, SDS protocol, and behavior evaluation time points. (**B**) Paw mechanical threshold by von Frey filament of SDS and no-SDS groups, days 0, 7, 11, and 21. (**C**) Paw thermal threshold by Hargreaves test of SDS and no-SDS groups, days 0, 7, 11, and 21. (**D**) Open-field test: total distance traveled in the arena (cm). (**E**) Mean body velocity of each animal (cm/s). (**F**) Maximum body acceleration (cm/s^2^). (**G**) Mean body mobility (%). Data represent the mean ± SEM. Two-way ANOVA followed by Bonferroni’s multiple comparisons test for (**A**,**B**). One-way ANOVA followed by Bonferroni’s multiple comparisons test for (**D**–**G**). * *p* < 0.05, ** *p* < 0.01, *** *p* < 0.001, **** *p* < 0.0001, ns means non-significant. n = 6 (SDS); n = 7 (no-SDS).

To assess whether chronic SDS can induce pain in juvenile mice, von Frey and Hargraves tests were performed at baseline, after four physical sessions (day 7), and after the end of the SDS protocol (days 11 and 21) (see experimental timeline in Figure 1A). Compared to the control group (no-SDS), stressed mice (SDS) demonstrated a slight decrease in the hind paw mechanical threshold on day 7, although this was not significant. From day 11, SDS animals showed a significant decrease in the paw threshold, which persisted until day 21 of the protocol, ten days after the last defeat session (Figure 1B, ***p < 0.001; *p < 0.05). Similar results were observed in the thermal stimulus test, where the SDS group demonstrated a significantly reduced hind paw withdrawal latency to the heat (~43 °C) compared to controls on days 11 and 21 (Figure 1C, ** *p* < 0.01; * *p* < 0.05). These findings show that SDS in juvenile male mice induces chronic mechanical allodynia and thermal hyperalgesia.

### 3.2. SDS Induces Long-Term Locomotor Changes and Short-Term Anxiety-like Behavior

To assess anxiety-like behavior and locomotor activity, we performed the open-field and light-and-dark tests after the last stress session (day 10) and seven days after SDS (day 17). As observed in Figure 1D, stressed animals showed a significant decrease in spontaneous activity behavior (less distance traveled in the arena) during the open-field test compared to the no-SDS group on both days evaluated (* *p* < 0.05, **** *p* < 0.00001). Corroborating this result, the mean velocity and mobility were also decreased in the SDS group (Figure 1E,F, * *p* < 0.05, **** *p* < 0.0001). Considering that mobility parameters indicate whether the mouse is highly mobile, moderately mobile, or immobile, our result suggests that chronic SDS resulted in a reduction in mobility, meaning less spontaneous motivation in the exploration of the arena, a profile that was not observed in the no-SDS group. Consequently, the decrease in mobility is reflected in the velocity measure since the speed is calculated by the distance traveled by the center, nose, or tail base point per unit of time. Interestingly, the max acceleration was significantly reduced on day 10 in SDS mice and reverted to a similar measurement of the control group on day 17 (Figure 1F, * *p* < 0.05). This behavioral profile uncovered that SDS in juvenile mice promoted persistent reduction in locomotor activity.

**Figure 2:**
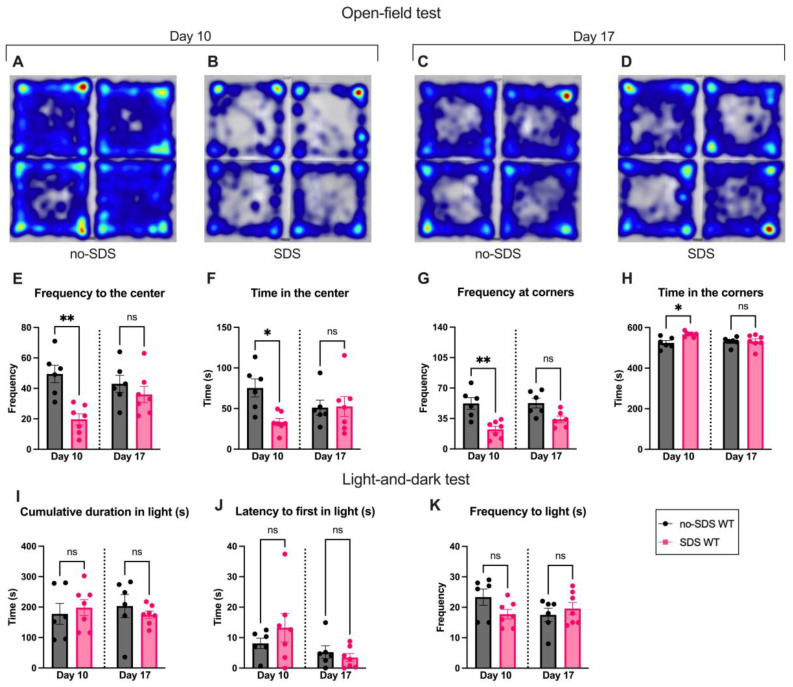
SDS reduces the spontaneous interest in exploring the arena on day 10. (**A**–**D**) Representative tracking heatmap image of four mice in the open-field arena. Color spectrum gradient: blue, low intensity; red, high intensity. (**A**) The no-SDS group and (**B**) the SDS group, on day 10. (**C**) The no-SDS group and (**D**) the SDS group, on day 17. (**E**) Frequency to the center of the arena. (**F**) Time spent in the center. (**G**) Frequency of visits to the corner of the arena. (**H**) Time spent in the corners. Ligh-and-dark test: (**I**) cumulative time spent in the bright chamber; (**J**) time until the first entrance of the bright chamber; (**K**) frequency of entries to the bright chamber. Data represent the mean ± SEM. One-way ANOVA followed by Bonferroni’s multiple comparisons test: * *p* < 0.05; ** *p* < 0.01; n = 6 (SDS); n = 7 (no-SDS); and ns means non-significant.

Furthermore, the open-field data showed that SDS triggers transient anxiety-like behavior. On day 10, the SDS group showed an anxiety-like phenotype, spending more time in the corners than in the center when compared to the no-SDS group (Figure 2A–H, * *p* < 0.05, ** *p* < 0.01). This pattern was not continued after 7 days of the SDS; stressed animals show a recovery effect of the anxious state, presenting similar data to the no-SDS group at day 17 (Figure 2A–H). Despite these findings, the SDS protocol did not promote significant changes in the behavior of the stressed group in the light-and-dark test on day 10 or 17 compared to no-SDS (Figure 2I–K). Thus, an aversion to the light was not observed after SDS sessions of male juvenile mice.

### 3.3. TLR4 Activation Is Increased in Spinal Cord and Dorsal Root Ganglia After SDS

**Figure 3:**
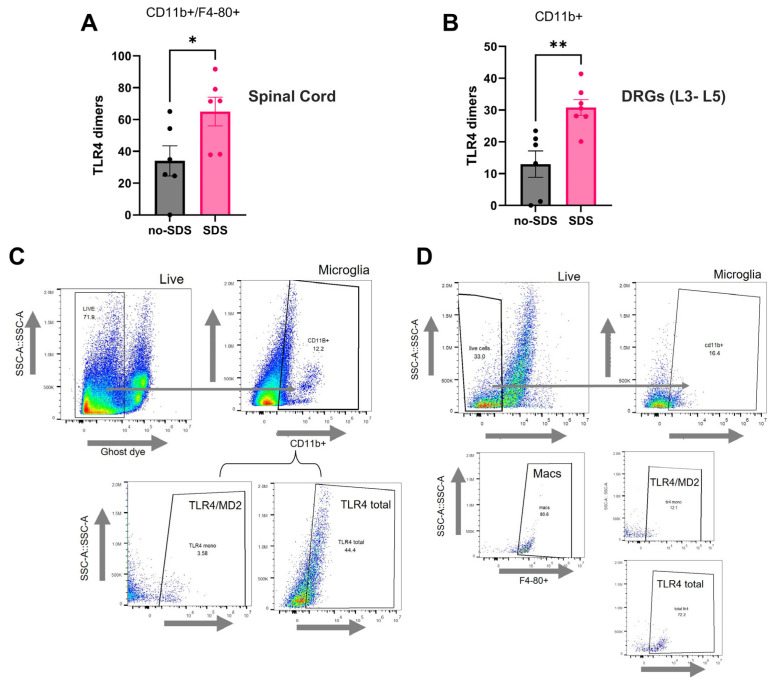
SDS increases TLR4 dimers of spinal cord microglia and DRG macrophages. (**A**) TLR4 dimers (%) in SDS and no-SDS groups of microglia (CD11b^+^ population) and (**B**) macrophages (CD11b^+^/F4-80^+^ population) on day 22 of the protocol. (**C**) Gating strategy for lumbar spinal cord cells, first live cells were gated (APC-Cy7 live/dead Ghost dye); second, microglia population (PercpCy 5.5-conjugated CD11b+), lastly—TLR4 monomers (PC7-conjugated TLR4/MD2) or TLR4 total (APC-conjugated total TLR4). (**D**) Gating strategy for dorsal root ganglia cells, first live cells were gated (APC-Cy7 live/dead Ghost dye); second, microglia population (PercpCy 5.5-conjugated CD11b+), third macrophages population (488-conjugated F4-80) and lastly—TLR4 monomers (PC7-conjugated TLR4/MD2) or TLR4 total (APC-conjugated total TLR4). Data represent mean ± SEM, *t*-test, SDS vs. no-SDS, * *p* < 0.05, ** *p* < 0.01, n = 6 (SDS), and n = 6 (no-SDS).

TLR4 activation was analyzed by flow cytometry by calculating dimerization (ratio monometers to total expression) in immune cells of the DRG, spinal cord (Figure 3A,B), and prefrontal cortex (Supplemental Figure S2) from the SDS and no-SDS groups. Significant changes were observed in the DRG and the spinal cord but not in the PFC. As seen in Figure 3, TLR4 dimerization was significantly increased in microglia cells (CD11b^+^) of the lumbar spinal cord in the SDS group compared to the no-SDS group (Figure 3A, * *p* < 0.05). Similarly, TLR4 dimerization was augmented on macrophage cells (CD11b^+^/F4-80^+^) of the DRG from stressed animals compared to non-stressed mice (Figure 3B, ** *p* < 0.01). Besides this, analyses from prefrontal cortex tissue in the flow cytometry or immunofluorescence assays did not show significant changes between the groups of animals regarding the number of Iba1^+^ cells (microglia activation marker) nor TLR4 dimerization in this population (Supplemental Figure S2A–D).

### 3.4. Genetic Ablation of TLR4 Expression Prevented Pain Phenotype Induced by SDS

**Figure 4:**
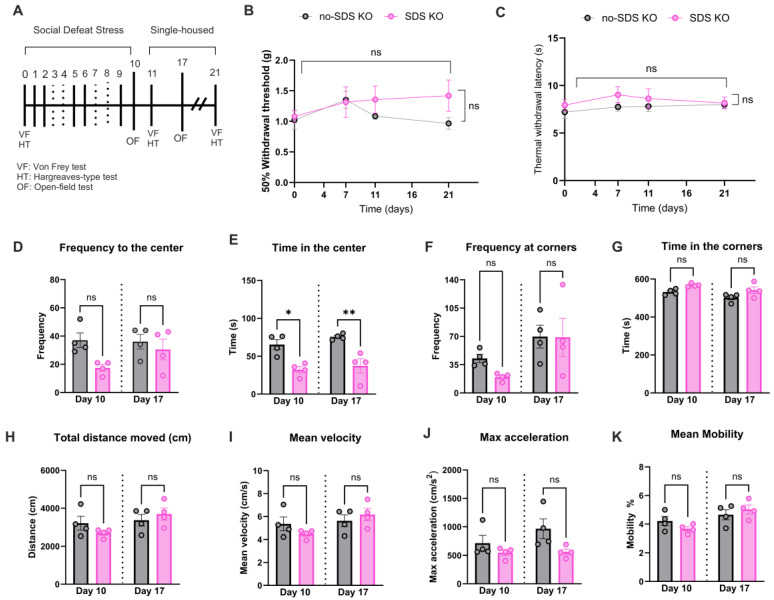
SDS did not promote the pain phenotype *Tlr4^-/-^* in mice. (**A**) Experimental timeline, SDS protocol, and behavior evaluation time points. (**B**) Paw mechanical threshold by von Frey filament of SDS and no-SDS KO mice at days 0, 7, 11, and 21. (**C**) Paw thermal threshold by Hargreaves test in SDS and no-SDS groups KO mice at days 0, 7, 11, and 21. Open-field test, in (**D**) total distance traveled in the arena (cm), in (**E**) time spent in the center, in (**F**) frequency of visit in corners of the arena, and (**G**) frequency of visit in the center area. (**H**–**K**) Locomotor activity parameters of SDS and no-SDS in KO mice. Data represent the mean ± SEM. Two-way ANOVA followed by Bonferroni’s multiple comparisons test for (**B**,**C**). One-way ANOVA followed by Bonferroni’s multiple comparisons test for (**D**–**K**). * *p* < 0.05, ** *p* < 0.01, n = 4 (SDS), n = 4 (no-SDS), and ns means non-significant.

To confirm the participation of TLR4 receptors in the development of chronic pain or the anxiety-like behavior phenotype in SDS, *Tlr4^-/-^* mice were subjected to the same protocol as the WT: six sessions of SDS, pain behavioral evaluations, and open-field tests (see experimental timeline in Figure 4A). As reflected by the von Frey readouts, SDS in *Tlr4^-/-^* mice did not show mechanical allodynia at any time point evaluated compared to no-SDS-KO (Figure 4B). A similar pattern was observed in thermal response after SDS in *Tlr4^-/-^* animals (Figure 4C). In the open-field test, the SDS-KO group decreased the time spent in the center of the arena in both times evaluated on days 10 and 17 (Figure 4E, * *p* < 0.05, ** *p* < 0.01). However, there were no significant changes regarding the frequency to the center and the corner and the time spent in the corners in SDS-KO compared to the no-SDS-KO group (Figure 4D,F,G). With respect to locomotor activity parameters, total distance, velocity, acceleration, and mean mobility, no significant differences were detected between groups (Figure 4H–K). Together, these results suggest that TLR4 receptor depletion prevented locomotor activity reduction and chronic pain development induced by SDS.

## 4. Discussion

The current work reveals, for the first time, that SDS induces pain sensitivity in juvenile mice by involving TLR4 dimerization in the dorsal root ganglia macrophages and spinal cord microglia. In addition, SDS promotes persistent locomotor activity reduction in stressed animals compared to non-stressed animals. The lack of TLR4 expression in knock-out mice prevented the development of chronic pain phenotypes and locomotor changes induced by SDS.

A physiological stress response can be triggered by physical (e.g., injury and inflammation) and, importantly, by psychological stressors, leading to system-wide, neurally initiated activation of pituitary and autonomic signaling [26]. As an example, the amygdala, in communication with the brain stem, promotes sympathetic adrenergic catecholamines, norepinephrine, and epinephrine release, resulting in typical stress symptoms: palpitation, high blood pressure, sweating, and pupil dilatation [4]. Long-lasting stress evokes hyperactivity in this system, lasting for an extended period even after the stress is discontinued [27]. In humans, chronic stressful experiences during childhood and adolescence (periods of intense social interaction) are shown to significantly impact mental health, resulting in anxiety and depression disorders, which can persist in adulthood [28]. Data from 2019 demonstrated that 970 million adults were found to be suffering from mental health disorders, and around 301 million suffer from anxiety, out of which 58 million were children and adolescents [29]. In parallel, studies proved that mental disorders caused by chronic stress exposure significantly affect the nociception pathways [27]. Animals subjected to stressful events presented an enhanced c-Fos expression in the spinal cord, an increase in glutamate, and a reduction in GABA, leading to enhanced central excitability [30,31,32]. In humans, chronic stress alongside psychological disorders proved to be the primary trigger for widespread musculoskeletal pain development known as fibromyalgia, one of the most challenging syndromes in clinics regarding pain management [33]. Collectively, these findings highlight the strong linkage between stress and pain processing. Here, we demonstrate that six sessions of SDS promote the chronic pain phenotype in juvenile male mice subjugated and intimated (physically and sensorially) by the retired aggressor CD1 mice. The SDS group presented a reduction in thermal and mechanical thresholds persisting up to 21 days, 10 days after the aggressor had been removed from the cage. Similarly, previous work showed that ten consecutive days of social stress in adult mice lead to persistent hyperalgesia, long-lasting depressive-like behavior, and social avoidance [9]. In our open-field data, however, SDS animals transiently exhibited depressive-like behavior; significant differences between the groups were only detected on day 10. Stressed mice presented lower distance traveled in the arena and fewer visits to the center area (a spot considered ‘unsafe’ and favorable to attacks) compared to non-stressed mice. SDS seems to reduce the spontaneous interest in exploring the arena, leading the animal to spend more time in the corners. This phenotype was, however, abolished after 7 days of social stress sessions, contrary to what was observed in the behavioral pain tests where allodynia persisted until day 21. Interestingly, the SDS protocol did not promote the natural aversion of stressed mice to brightly illuminated areas on our light-and-dark test. These findings indicate that juvenile mice might exhibit different anxiety behavior patterns compared to adults, recovering their interest in exploring the arena after a few days of SDS. Nevertheless, in another study, stressed juvenile mice did not show a significant difference in exploring the arena compared to the control group in the open-field test, although they exhibited an increase in immobility behavior during tail-suspension and forced swimming tests [34]. Similar changes in locomotor activity due to stress were found in our results. The SDS group had reduced velocity and acceleration on both time points analyzed, which was reflected in lower body mobility than the non-stressed group. Accordingly, it has been reported that neonatal maternal separation-induced stress reduces exploratory activity in adult rats [35]. This is unlike the chronic restraint stress model, examined for 5 days in adult rats, which was shown to promote an increase in exploratory behavior [36]. Even though there are conflicting results, this evidence suggests that species, age, and type or duration of the stress protocol can lead to distinct anxiety-like behavior parameters in rodents.

Recent work has indicated that chronic stress facilitates nociceptive transmission through neuroinflammatory signaling involving microglia-mediated neuronal remodeling [37]. TLR4 proteins are abundantly expressed in spinal and brain microglia and DRG macrophages as monomers in cholesterol-rich lipid rafts distributed on the cell surface [38]. Once activated, they form TLR4 homodimers and an aggregation of these lipid rafts. This activation and raft aggregation results in two events. First, TLR4 dimerization triggers downstream signaling via phosphorylation of secondary messengers, activation of the cellular inflammasome, and transcriptomic changes in the sensory neuron, resulting in the initiation of an afferent hyperexcitable state [39]. Second, the TLR4 rafts express other receptors, such as N-methyl-d-aspartate (NMDA-r) [40], transient receptor potential vanilloid 1 (TRPV1) [12,13,41], and channels such as voltage-gated sodium channels [42]. TLR4-raft activation aggregates these channels and receptors, leading to a significant increase in membrane excitability. An in vivo TLR4 blockade attenuates hyperalgesia triggered by intrathecal LPS and neuropathic pain induced by nerve constriction injury or by chemotherapy drugs [17,43,44]. According to our previous findings and in line with other work, TLR4 is a microglial receptor critically responsible for developing and maintaining a pain phenotype [12,13,17,18,43,44]. In our study, TLR4 dimerization in spinal microglia and dorsal root ganglia macrophages was associated with the pain phenotype induced by social stress in juvenile mice compared to non-stressed controls up to 10 days after the end of the SDS protocol. Previously, using the social defeat stress model, a group found an increase in microglial activation and overexpression of inflammatory genes such as TLR4 in the spinal cord, which was associated with stress-induced hyperalgesia [37]. Accordingly, in this work, the global genetic depletion of TLR4 prevented the development of mechanical allodynia or thermal hyperalgesia, and no changes were observed in locomotor activity parameters, such as velocity, acceleration, or mean mobility in SDS-KO mice compared to no-SDS KO. However, a partial anxiety-like behavior was observed in KO mice subjected to SDS. These animals spent less time in the center of the arena on days 10 and 17 of the protocol compared to the non-stressed KO group. These findings indicate that TLR4 seems to be more related to the pain phenotype than the anxiety disorder promoted by SDS in juvenile mice.

TLR4 ligands, such as HMGB1 (chromatin protein high-mobility group box 1), are shown to be extracellularly released by immune cells such as macrophages and microglia under a stress state [45] into the extracellular space. Through TLR (including TLR4) activation, these ligands induced NF-κB signaling and proinflammatory cytokine production that affect the nociceptors [46]. Considering that the DRG is located outside the blood–brain barrier [47], circulating products released by stress from small amines (noradrenalin) to larger proteins (hormones and cytokines) can easily reach the cells of DRG, which can reflect changes in TLR4 signaling. In addition, bulbospinal pathways are recognized to regulate dorsal horn excitability. Of note, serotonergic pathways facilitate pain signaling, activating dorsal horn neurons and microglia [48,49,50]. Serotonin receptor activation increases afferent terminal excitability [51] and activates DRG neurons [52]. Consistent with these observations, stress, as with models of conditioned fear mediated by supraspinal integration and learning, produces a profound change in spinal processing through the activation of bulbospinal serotonin projections [53]. These considerations jointly point to the possibility that higher order processes can initiate changes in downstream neuraxial signaling and excitability.

In contrast to the DRG and the spinal cord findings, we unexpectedly observed no significant changes in SDS male mice at the prefrontal cortex (PFC) regarding TLR4 dimerization or the quantity of Iba1^+^ cells (microglia activation). In a study by Yang and collaborators (2024), adult female and male mice subjected to 28 days of stress also showed no difference in the number of Iba1^+^ microglial cells in the PFC but instead showed changes in morphology and increased phagocytic activity of microglia, a change observed only in females and not in males. Moreover, an increase in microglia genes linked to the TLR4 signaling pathway was detected in stressed females [54]. Additionally, adult male mice exposed to fear conditioning protocols presented increased Iba1^+^ cells on medial PFC on days 1 and 3 but no significant changes were observed on days 7 or 14; even after remote recall, the increased microglia activity in PFC lasted for one hour [55]. Sexual dimorphism, the mice’s age, and tissue collection time can explain the absence of changes in PFC from SDS juvenile male mice in our experiments. Further research is necessary to better understand the role of microglia and TLR4 activity at PFC in the pain modulation of juvenile mice after social stress due to the complexity of this structure.

## 5. Limitations of the Study

Our social defeat stress work did not extend to C57BL/6J female juvenile mice since male CD1 aggressors did not present aggressive or intimidating behavior; instead, they initiated mate bonding. This caveat makes it challenging to investigate sexual dimorphism in pain phenotypes using this stress paradigm. However, a key advantage of SDS is that it more accurately mimics the modern human environment that features high levels of social stressors, unlike physical restraint and forced swim models, which impose stressors rarely encountered in real life for humans.

Although our work proved the involvement of TLR4 activity in pain induced by social stress using a genetic approach, pharmacological interventions such as TLR4 inhibitors, conditional knockdowns, or viral vectors to modulate TLR4 expression could further elucidate its role in stress-induced pain. In addition, characterization of the role of descending modulatory pathways is also pertinent to understanding the central mechanisms that lead to spinal sensitization induced by chronic stress. Future studies could build upon these findings to refine therapeutic approaches.

## 6. Conclusions

SDS in juvenile male mice promotes chronic pain phenotypes and persistent locomotor activity reduction. These outcomes are related to the dimerization of TLR4 receptors in spinal microglia and dorsal root ganglia macrophages. The pain phenotype caused by SDS was prevented by the genetic ablation of TLR4 receptors in stressed mice.

## Data Availability

The data presented in this study are available upon request from the corresponding author. The data presented in this work were part of a master’s thesis in Biology at the University of California, San Diego, from Alisa Panichkina.

## Supplementary Materials

The following supporting information can be downloaded at https://www.mdpi.com/article/10.3390/cells14050350/s1: Figure S1: Experimental setup; Figure S2: PFC molecular analysis.
