# Supplementary material for: Chronic Pain Induced by Social Defeat Stress in Juvenile Mice Depends on TLR4
Source: Cells. 2025 Feb 27;14(5):350. doi: 10.3390/cells14050350 (PMC11898947; doi:10.3390/cells14050350)
Supplement: Supplementary file 1 [file cells-14-00350-s001.zip › cells-3459041-supplementary.pdf]

Supplementary Materials:

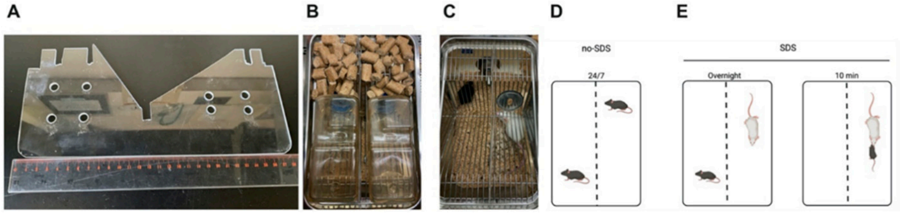

Figure S1. Experimental setup. **(A)** A perforated plexiglass divider used to separate CD1 and C57. **(A)** Cage setup during SDS protocol with or without the water bottle and food. **(B-C)** Representative diagram of SDS group cage setup (white mouse represents CD1 and black mouse C57). **(D)** Representative diagram from the control group (one black mouse C57 on each side). **(E)** Representative diagram of SDS group: left side – sensorial contact; right side- physical contact with aggressor CD1. Images were generated by our group, and diagrams by Biorender program.

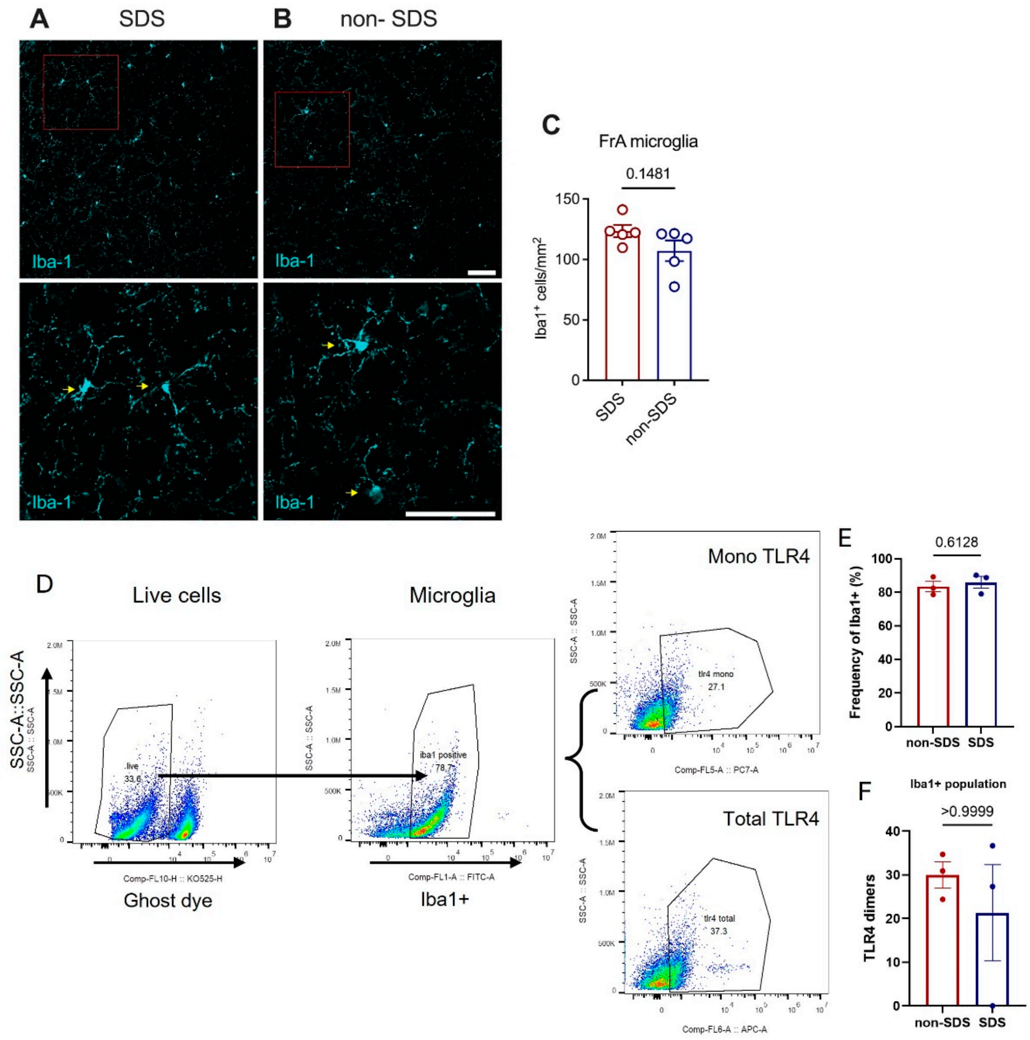

Figure S2. PFC molecular analysis. Representative images from the immunofluorescence assay of PFC. Upper: Iba1+ cells (Microglia-cyan color) of non-SDS **(A)** and SDS mice **(B)**. Lower: high magnification images of the boxes outlined in the upper panel, yellow arrows indicating Iba1+ cells, microglia. **(C)** Quantification of Iba1+ cells, no significant difference between the groups. Scale bar=50 mm. (n=5). **(D)** Flow cytometry gating strategy and population of Iba1^{+}/TLR4/MD2^{+} (Mono)/total TLR4^{+} of PFC. **(E)** TLR4 dimers from PFC of SDS and no-SDS mice, no significant difference between the groups (n=3). Unpaired t-test.
